# Exploring clinical specialists’ perspectives on the future role of AI: evaluating replacement perceptions, benefits, and drawbacks

**DOI:** 10.1186/s12913-024-10928-x

**Published:** 2024-05-09

**Authors:** Muhammad Daniyal, Moiz Qureshi, Roy Rillera Marzo, Mohammed Aljuaid, Duaa Shahid

**Affiliations:** 1 Department of Statistics, Faculty of Computing, Islamia University of Bahawalpur, Bahawalpur, Pakistan; 2 Government Degree College, TandoJam, Hyderabad, Sindh Pakistan; 3 Faculty of Humanities and Health Sciences, Curtin University, Malaysia, , Miri, Sarawak Malaysia; 4 Jeffrey Cheah School of Medicine and Health Sciences, Global Public Health, Monash University Malaysia, Subang Jaya, Selangor Malaysia; 5 Department of Health Administration, College of Business Administration, King Saud University, Riyadh, Saudi Arabia; 6 Hult International Business School, 02141 Cambridge, MA USA

**Keywords:** AI, Machine learning, Medical imaging, Curriculum, Privacy issue, Clinical specialists

## Abstract

**Background of study:**

Over the past few decades, the utilization of Artificial Intelligence (AI) has surged in popularity, and its application in the medical field is witnessing a global increase. Nevertheless, the implementation of AI-based healthcare solutions has been slow in developing nations like Pakistan. This unique study aims to assess the opinion of clinical specialists on the future replacement of AI, its associated benefits, and its drawbacks in form southern region of Pakistan.

**Material and methods:**

A cross-sectional selective study was conducted from 140 clinical specialists (Surgery = 24, Pathology = 31, Radiology = 35, Gynecology = 35, Pediatric = 17) from the neglected southern Punjab region of Pakistan. The study was analyzed using χ^2^ - the test of association and the nexus between different factors was examined by multinomial logistic regression.

**Results:**

Out of 140 respondents, 34 (24.3%) believed hospitals were ready for AI, while 81 (57.9%) disagreed. Additionally, 42(30.0%) were concerned about privacy violations, and 70(50%) feared AI could lead to unemployment. Specialists with less than 6 years of experience are more likely to embrace AI (*p* = 0.0327, OR = 3.184, 95% C.I; 0.262, 3.556) and those who firmly believe that AI knowledge will not replace their future tasks exhibit a lower likelihood of accepting AI (*p* = 0.015, OR = 0.235, 95% C.I: (0.073, 0.758). Clinical specialists who perceive AI as a technology that encompasses both drawbacks and benefits demonstrated a higher likelihood of accepting its adoption (*p* = 0.084, OR = 2.969, 95% C.I; 0.865, 5.187).

**Conclusion:**

Clinical specialists have embraced AI as the future of the medical field while acknowledging concerns about privacy and unemployment.

**Supplementary Information:**

The online version contains supplementary material available at 10.1186/s12913-024-10928-x.

## Introduction

Artificial intelligence (AI) generally applies to computational technologies that emulate mechanisms assisted by human intelligence, such as thought, deep learning, adaptation, engagement, and sensory understanding [1, 2]. Some devices can execute a role that typically involves human interpretation and decision-making [3, 4]. These techniques have an interdisciplinary approach and can be applied to different fields, such as medicine and health. AI has been involved in medicine since as early as the 1950s when physicians made the first attempts to improve their diagnoses using computer-aided programs [5, 6]. Interest and advances in medical AI applications have surged in recent years due to the substantially enhanced computing power of modern computers and the vast amount of digital data available for collection and utilization [7]. AI is gradually changing medical practice. Several AI applications in medicine can be used in a variety of medical fields, such as clinical, diagnostic, rehabilitative, surgical, and predictive practices. Another critical area of medicine where AI is making an impact is clinical decision-making and disease diagnosis. AI technologies can ingest, analyze, and report large volumes of data across different modalities to detect disease and guide clinical decisions [8–10].

It is possible that the widespread use of advanced technologies and the imminent challenges of AI could pose a threat to physicians’ jobs and experts feel that the role of physicians will customarily become a joint team effort between physicians and machines [4]. In a survey involving 791 psychiatrists from advanced countries, 83% of participants expressed their opinions about the future capability of technology to provide care, while they also showed concerns about the potential replacement of their roles [11]. Conversely, a study with radiologists showed that 77% displayed favorable attitudes towards AI implementation and 89% exhibited a lack of concern regarding job displacement [12]. Neurosurgeons, too, embraced AI, with 60% utilizing it for outcome prediction, and a staggering 90% of physicians across diverse specialties in Germany anticipated AI integration in the future of medicine [11, 12]. A global survey of pathologists revealed widespread acceptance of AI, as only 17.6% showed their apprehensions about job security [13]. This was also observed in a Saudi Arabian study, where physicians, nurses, and technologists expressed notable concerns about their future employability [14]. The optimism persisted in a study among neurosurgeons, reinforcing the anticipation of AI as a crucial component in the future of medicine [15]. Concerning patient perspectives on AI in clinical practice, a prevailing preference for human physicians over AI was observed [16–19]. This inclination may be attributed to the proven value of AI algorithms in aiding radiologists to detect abnormalities in medical images, categorize conditions, hypothesize about underlying health issues, suggest suitable procedures, and interpret results [20, 21]. The capabilities of AI extended to dermatology, where it demonstrated robust diagnostic imaging and assessments for various dermatological pathologies. In ophthalmology, AI emerged as a valuable tool for identifying and understanding eye problems, aiding doctors in recognizing issues within the retina and other ocular structures [22–25]. The exploration of physicians’ perspectives on AI in the Arab world revealed optimism among doctors in Oman, where a study with 300 doctors and 750 medical students unveiled a positive outlook on AI’s future role, coupled with minimal concerns about job displacement [26]. A similar study in the United Arab Emirates involving 119 individuals skilled in conducting X-rays and 34 specialized X-ray doctors demonstrated that 86% did not envision AI playing a role in X-ray procedures. Furthermore, 64% expressed a lack of concern about AI negatively impacting their careers in this specific domain [27].

Pakistan, with a population exceeding 220 million people, is facing distinctive healthcare challenges arising from its diverse demographics, varying economic conditions, and geographical disparities. Ranked as the fifth-most populous country globally, Pakistan exhibits a populace dispersed across urban and rural landscapes, with healthcare infrastructure discrepancies evident between urban centers and remote rural areas. Besides these challenges, the country has initiated an AI program under a presidential initiative, signifying a forward-looking approach [28]. However, the implementation of AI in the health sector encounters several hurdles, especially in medical healthcare. Recent advancements in AI application in Pakistan prompt attention to the need for rigorous evaluation, particularly through randomized controlled trials (RCTs) to establish effectiveness across diverse healthcare settings. A systematic review uncovered a deficiency of RCTs involving AI within the Pakistani healthcare system [29]. Furthermore, the absence of a published dataset for retinal images poses a notable challenge for doctors seeking to train AI models, limiting the integration of AI into routine medical practices [30].

Against this backdrop, to contribute to the existing literature on the impact of AI in Pakistan, this study aims to delve into the perspectives of clinical experts situated in the southern region of Punjab. This region, like numerous others in Pakistan, grapples with challenges related to healthcare infrastructure [31].

The study seeks to explore how AI might influence the daily routines of healthcare professionals in this region, examining the anticipated benefits and potential implications for patient confidentiality and job stability. Moreover, the findings of this study could have broader implications for healthcare systems in developing countries facing similar challenges. By examining the perceived benefits and drawbacks of AI adoption from the perspective of healthcare professionals in Pakistan, this study contributes to the global discourse on the role of AI in healthcare and provides valuable insights into AI strategies to meet the specific needs of diverse healthcare contexts. The impact of AI on healthcare in Pakistan is greater than in other countries due to its unique healthcare challenges, recent initiatives in AI implementation, and the potential for transformative change in addressing longstanding healthcare disparities. Understanding the perspectives of clinical experts in regions like southern Punjab not only informs local strategies but also contributes to broader discussions on AI adoption in healthcare systems worldwide.

By focusing on the southern region of Punjab, the research aims to find the answers to the following questions;

- To what extent do clinical specialists in southern Punjab, Pakistan, perceive AI as a replacement for their knowledge and expertise?
- How do clinical specialists in southern Punjab, Pakistan, perceive the potential benefits and drawbacks of AI in their practice and the broader healthcare landscape?

This study is structured as follows: The second section presents the materials and methods, encompassing the study design, participant demographics, and ethical considerations. The third section details the data analysis, followed by the presentation of results regarding AI knowledge as a replacement, its benefits, drawbacks, and acceptance. Finally, the study ends with a discussion and conclusion section, where it reflects on its findings and acknowledges the possible limitations of the study.

## Materials and methods

### Design and participants

A cross-sectional survey was conducted during the period from September to December 2022, in the Southern Punjab region, Pakistan. The study’s inclusion criteria were carefully designed to ensure a representative sample of clinical specialists who could provide valuable insights into the impact of AI on healthcare in Pakistan, particularly in the southern region of Punjab. To be eligible for participation, participants should be practicing clinical specialists in various fields of healthcare, mainly surgery, pathology, radiology, gynecology, and pediatrics. Clinical specialists of any gender were required to have a minimum of five years of experience following their graduation from medical school. With a minimum of five years of post-graduate experience, participants were likely to have encountered various challenges and advancements within the healthcare sector, providing them with valuable insights into the potential implications of AI adoption. Furthermore, by not imposing any gender-specific criteria, the study aimed to promote inclusivity and diversity in its participant pool.

The self-structured questionnaire was designed and disseminated to professional networks of doctors through social media platforms. Two independent professional investigators were responsible for the questionnaire’s development, and any discrepancies were resolved through discussion. A senior faculty member reviewed and validated the questionnaire. Convenience sampling was employed to select the sample, and participants were queried about demographic details (age, gender, level of qualification, affiliated institute), their knowledge about AI and its applications, as well as their perceptions and practices regarding AI. Before the formal survey, a pilot survey was conducted with a limited number of respondents to assess the online questionnaire’s usability and technical functionality. Respondents had the opportunity to review and modify their answers. Duplicate entries were meticulously eliminated from the analysis, and only fully completed questionnaires were included. The inclusion criteria specified that respondents must be medical personnel who completed the survey, while non-medical responders and incomplete surveys were excluded from the analysis. No personal information was collected or stored. After that, the questionnaire was tested on 15 clinical specialists as part of a pilot study to confirm its reliability. The tool’s internal consistency of the used sub-scales was shown by Cronbach’s alpha values, (AI knowledge as replacement = 0.703, benefits and drawbacks = 0.719)

Based on an assumed effective response distribution of 80% and a total population size of 315, the estimated sample size required was 150 participants. Ultimately, we obtained a sample of 140 participants for the survey. The survey was conducted online through the utilization of Google Forms. Before their participation, the participants received detailed information about the study’s objectives and procedures. The participants were asked the questionnaire based on demographic characteristics, their work experience, and clinical specialties. The questionnaire was further categorized into two portions, one included portion consisted of nine questions on AI’s different roles as replacements of documentation, clinical care to patients, suggesting medication, conducting a physical examination, diagnosis, and patient history (for data analysis, Possible = 1, Not Possible = 2, Maybe Possible = 3) and second portion consisted of eight questions about AI benefits and drawbacks including the concerns about violation of patients privacy, unemployment issue, reducing the work and paper burden, support for specialists and part of curriculum (For the data analysis, Yes = 1, No = 2, Maybe = 3). Informed e-written consent, along with the terms and conditions, was obtained from each participant. Participation in the survey was entirely voluntary, and participants had the freedom to withdraw from the study at any point without any obligation.

### Ethical considerations

This study adhered to the Declaration of Helsinki for the recruitment of human subjects and was approved by the Ethics Committee of the Punjab Health Department of District Multan, Pakistan. Informed consent was obtained from all the participants and/or their legal guardians.

## Statistical analysis

Statistical analyses were performed using SPSS software version 27, and data visualization was performed using JASP software version 14. Descriptive statistics, such as means with standard deviations, were used to analyze continuous variables. Categorical variables were presented as frequencies with percentages. The association between different categorical variables was assessed by χ2 - test of association. Acceptance of AI as future was selected as the dependent variable while independent variables in the model are “Age”, “Experience”, “Gender”, " AI’s Knowledge as a replacement in future tasks”, " AI’s drawbacks and benefits”, “Acceptance of AI by the hospital in future”. We computed odds ratios (OR) using multinomial logistic regression to present the relationship between dependent and independent categorical variables. The results included 95% confidence intervals (C.I) and *p*-values.”

## Results

The study involved an extensive data collection process from a diverse group comprising 140 participants, consisting of clinical specialists in their fields with varying ages and experience levels. Among the respondents, there were more male participants (80 individuals, representing 57.1%) compared to female participants 60(42.9%). The age distribution of the participants was also analyzed, with 68 (48.6%) being above the age of 40, 42 (30%) of study participants falling below the age of 35, and 30 (21.4%) belonging to the age range of 36 to 40. Furthermore, the participants’ experience levels demonstrated considerable heterogeneity, as 59(42.1%) reported having less than six years of experience, 55(39.3%) had more than 11 years of experience, and 26(18.6%) fell within the 7 to 11 years experience range. The study encompassed multiple departments, with Surgery representing 24(17.1%), Pathology accounting for 31(22.1%), Radiology including 35(25%), Gynecology comprising 33(23.6%), and Pediatrics comprising 17(12.1%) of the participants. Surprisingly, only 34(24.3%) expressed a belief that hospitals were ready to embrace AI-driven diagnostic tools as a new tool in the field of diagnostics, while a substantial majority of 81(57.9%) displayed doubt about it, stating that hospitals were not adequately prepared for AI implementation as diagnostics. A smaller but still noteworthy proportion of 25(17.9%) were cautiously optimistic, asserting that hospitals were partially ready for AI integration. Table 1 provides a comprehensive breakdown of the socio-demographic characteristics of the participants, as well as their knowledge and acceptance levels concerning AI (Table 1).

**Table 1:** Socio-demographic characteristics and AI knowledge and acceptance (*N* = 140)

Variables		N	%
Age (40.77 ± 6.79)	Less than 35	42	30.0%
	36–40	30	21.4%
	Above 40	68	48.6%
Experience (10.84 ± 6.49)	Less than 6	59	42.1%
	7–11	26	18.6%
	More than 11	55	39.3%
Gender	Male	80	57.1%
	Female	60	42.9%
Department	Surgery	24	17.1%
	Pathology	31	22.1%
	Radiology	35	25.0%
	Gynecology	33	23.6%
	Pediatrics	17	12.1%
Do you think hospitals are ready to accept AI-driven diagnostic tools as a new tool	Yes	34	24.3%
	No	81	57.9%
	To some extent	25	17.9%
Do you think AI is the future?	Yes	71	50.7%
	No	41	29.3%
	To some extent	28	20.0%
Knowledge about AI	Poor	21	15.0%
	Average	41	29.3%
	Above average	54	37.1%
	Excellent	24	16.4%

In terms of participants’ perception of AI’s role in the future, a substantial 71(50.7%) of respondents believed that AI held a vital position in shaping the future of healthcare. Conversely, 41(29.3%) of participants expressed doubts about AI being the future, while 28(20.0%) thought AI would play a role to some extent. The survey also assessed the participants’ knowledge of AI, with the results showcasing diverse levels of understanding. A considerable proportion, 54(37.1%), displayed above-average knowledge, followed by 41(29.3%) with average knowledge, and 24(16.4%) with excellent knowledge about AI. On the other hand, 21(15.0%) of participants had a poor understanding of AI.

## AI knowledge as a replacement for clinical specialists

This sub-scale consisted of nine questions consisting of AI’s different roles as replacements of documentation, clinical care to patients, suggesting medication, conducting a physical examination, diagnosis, and patient history (for data analysis, Possible = 1, Not Possible = 2, Maybe Possible = 3) AI’s most prominent role in documenting patient information, including health records and patient history, is likely to be in Surgery 12(8.6%) and Pathology 9(6.4%), while Radiology and Gynecology show lower percentages at 4(2.9%) and Pediatrics at 3(2.1%) (*p* = 0.018**). However, in terms of providing good patient care in the future, the utilization of AI appears relatively low across all specialties, ranging from 2(1.4%) to 7(5.0%). Notably, Radiology stands out as the specialty where AI’s possible involvement in suggesting personalized medication based on patient history is most prevalent, with a significant percentage of 13.6% (*p* = 0.011).

**Table 2:** Opinion of clinical specialists about the “Possible” AI can replace the experts to perform tasks in the future

Tasks that AI can Perform in the Future as a replacement for Specialists	Surgery	Pathology	Radiology	Gynecology	Pediatrics	χ^2^(*p*-value)
	n(%)	n(%)	n(%)	n(%)	n(%)	
Documentation about patients (Health records, patient history, etc.)	12(8.6%)	9(6.4%)	4(2.9%)	4(2.9%)	3(2.1%)	18.423 (0.018**)
Providing good care to patients	7(5.0%)	3(2.1%)	7(5.0%)	7(5.0%)	2(1.4%)	12.265 (0.140)
Suggesting personal medication based on the patient’s history	11(7.9%)	4(2.9%)	19(13.6%)	10(7.1%)	2(1.4%)	19.854 (0.011**)
Evaluation of the patients for treatments	9(6.4%)	9(6.4%)	8(5.7%)	10(7.1%)	6(4.3%)	2.755 (0.949)
Examine patient information to establish prognoses	12(8.6%)	7(5.0%)	7(5.0%)	10(7.1%)	7(5.0%)	10.127 (0.256)
Employ advanced techniques to detect instances of self-harmful behavior in patient information	15(10.7%)	6(4.3%)	5(3.6%)	11(7.9%)	6(4.3%)	20.430 (0.001***)
Conduct a comprehensive physical examination, encompassing a detailed mental status assessment	15(10.7%)	9(6.4%)	1(0.7%)	3(2.1%)	5(3.6%)	34.628 (0.000***)
Utilize advanced methods to discern potential indications of criminal behavior or assault in patient information	8(5.7%)	15(10.7%)	9(6.4%)	7(5.0%)	5(3.6%)	25.544 (0.001***)
Conduct patient interviews across diverse settings to gather their comprehensive medical history	8(5.7%)	10(7.1%)	11(7.9%)	12(8.6%)	4(2.9%)	16.045 (0.042**)

n(%) of the “Possibly” Category

if *p* < 0.05, the results will be declared significant

**significant

***highly significant

**Fig. 1:**
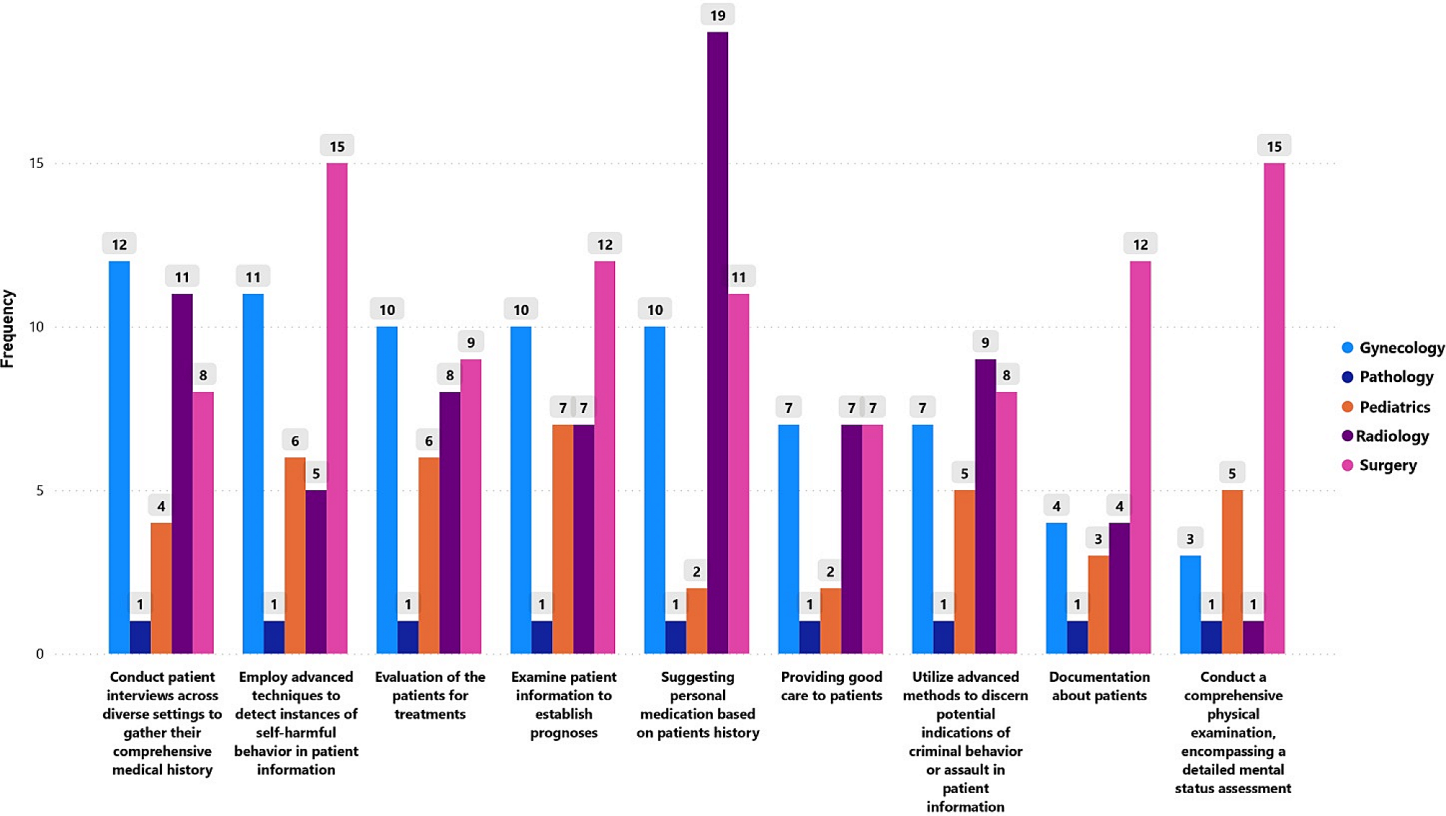
AI can perform future tasks as replacements for specialists (“Possible” opinion by specialist)

When evaluating patients for treatments using AI, the task seems to be evenly distributed across all specialties, with no significant differences observed (*p*-value = 0.949). Similarly, AI’s possible role in examining patient information to establish prognoses shows consistent percentages across specialties, with no significant differences (*p*-value = 0.256). A noteworthy finding is that it is possible that in the future AI can be frequently employed to detect instances of self-harmful behavior in patient information, particularly in Surgery with the highest percentage at 10.7%. The *p*-value of 0.001*** highlights a highly significant association between AI use and the detection of self-harmful behavior. Likewise, AI’s “possible” use to discern potential indications of criminal behavior or assault in patient information varies significantly among specialties (*p* = 0.001***). Conducting a “possible” comprehensive physical examination with a detailed mental status assessment using AI is most prevalent according to the Surgery and Gynecology specialists, with percentages of 10.7% and 3.6%, respectively (*p* = 0.000**). Finally, AI’s “possible” involvement in conducting patient interviews across diverse settings to gather comprehensive medical histories showed significantly varying percentages across specialties, with the highest in Surgery at 10.7% (*p* = 0.042) (Table 2; Fig. 1).

## AI’s benefits and drawbacks

**Fig. 2:**
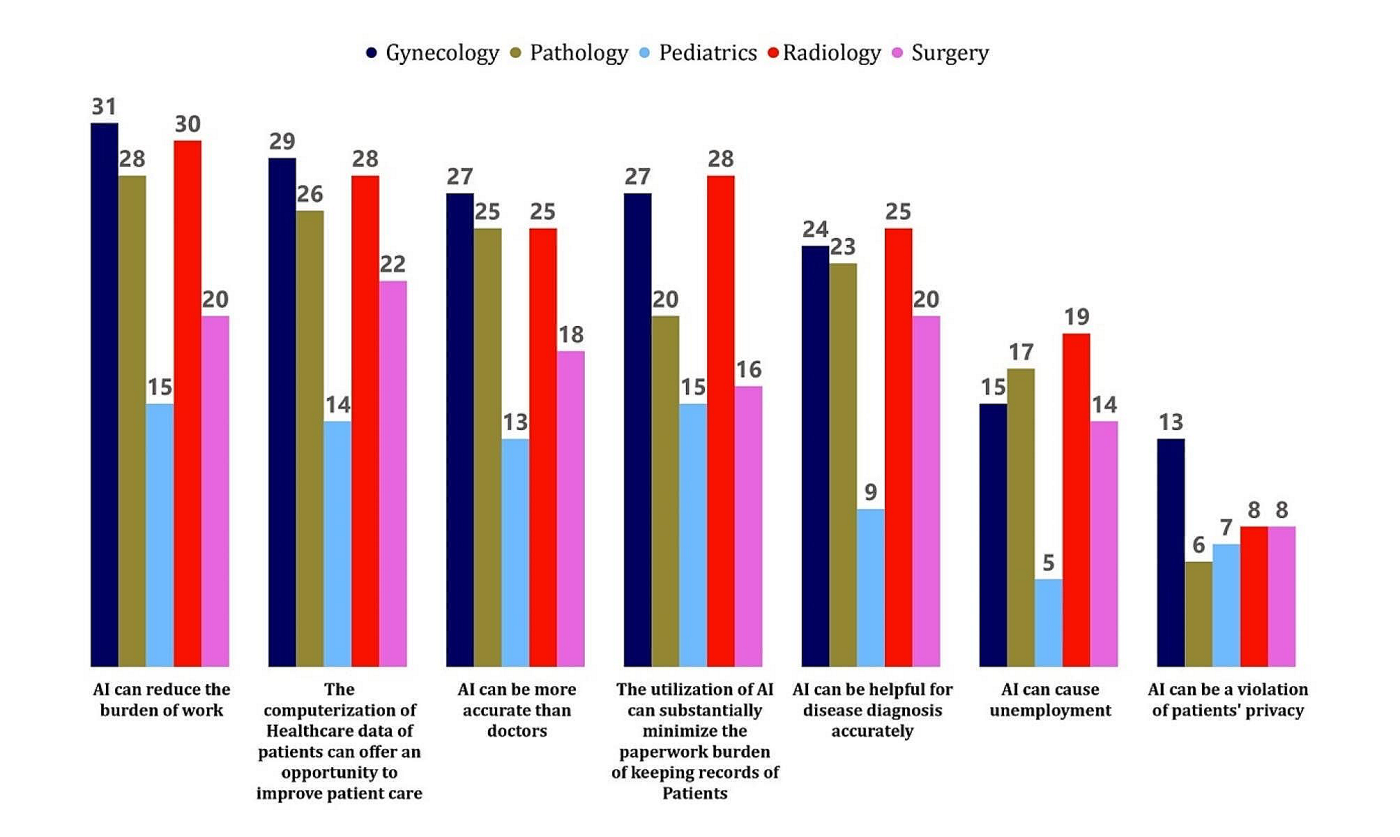
Opinion of specialists (“YES”) about the benefits and drawbacks of AI (*N* = 140)

This sub-scale consisted of eight questions about AI benefits and drawbacks including the concerns about violation of patients’ privacy, unemployment issues, reducing the work and paper burden, help for specialists, and part of the curriculum (For the data analysis, Yes = 1, No = 2, Maybe = 3). The study revealed that 42(30.0%) of them expressed the view that AI can be a violation of patients’ privacy. Looking at the responses from different departments, gynecology had the highest number of specialists who agreed with this statement, with 13(9.3%) respondents. On the other hand, 62(44.3%) specialists disagreed with the statement that AI can violate patients’ privacy. The majority of respondents in each department fell under this category, with Pathology having the highest number of specialists, 16(11.4%), who disagreed, followed by Radiology with 15 (10.7%) and Gynecology with 14 (10.0%) respondents. 36(25.7%), were unsure and responded with “maybe.” (Table 3; Fig. 2).

**Table 3:** Opinion of specialists (“YES”) about the benefits and drawbacks of AI (*N* = 140)

AI Benefits and Drawbacks	Clinical Specialists					χ^2^(*p*-value)
	Surgery	Pathology	Radiology	Gynecology	Pediatrics	
	n(%)	n(%)	n(%)	n(%)	n(%)	
AI can be a violation of patients’ privacy	8(5.7%)	6(4.3%)	8(5.7%)	13(9.3%)	7(5.0%)	7.489 (0.485)
AI can cause unemployment	14(10%)	17(12.1%)	19(13.6%)	15(10.7%)	5(3.6%)	13.517 (0.095*)
The computerization of Healthcare data of patients can offer an opportunity to improve patient care	22(15.7%)	26(18.6%)	28(20.0%)	29(20.7%)	14(10.0%)	5.827 (0.667)
The utilization of AI can substantially minimize the paperwork burden of keeping records of Patients	16(11.4%)	20(14.3%)	28(20.0%)	27(19.3%)	15(10.7%)	11.878 (0.157)
AI can reduce the burden of work	20(14.3%)	28(20%)	30(21.4%)	31(22.1%)	15(10.7%)	7.931 (0.440)
AI can be helpful for disease diagnosis accurately	20(14.3%)	23(16.4%)	25(17.9%)	24(17.1%)	9(6.4%)	6.201 (0.625)
AI can be more accurate than doctors	18(12.9%)	25(17.9%)	25(17.9%)	27(19.3%)	13(9.3%)	6.937 (0.543)
AI can be part of the curriculum of Medical students	16(11.4%)	24(17.1%)	30(21.4%)	27(19.3%)	11(7.9%)	14.069 (0.080*)

n(%) of “Yes” Category

*p < 0.10 (significant)

Additionally, 70(50%) of the clinical specialists expressed the opinion that AI can cause unemployment in the healthcare sector. Among the departments, Radiology had the highest number of specialists who agreed with this statement, with 19(13.6%) respondents. This was followed by Pathology with 17(12.1%) specialists agreeing, and Surgery with 14 (10%) specialists in agreement. Gynecology and Pediatrics departments had 15 (10.7%) and 5 (3.6%) specialists, respectively, who believed that AI could lead to unemployment in the field.

51(36.4%) specialists disagreed with the statement that AI can cause unemployment. Among the study participants, 119(85%) expressed the view that the computerization of AI of healthcare data by AI of patients can indeed offer an opportunity to improve patient care. Among the departments, Gynecology had the highest number of specialists who agreed with this statement, with 29 (20.7%) respondents. In contrast, 21(15%) specialists disagreed with the statement that computerization can improve patient care. Moreover, 106(75.7%) expressed the view that the utilization of AI has indeed substantially minimized the paperwork burden of keeping patient records. While 101(72.1%) expressed the view that AI can indeed be helpful for disease diagnosis accurately. Among the clinical specialists, radiologists specialists showed the highest numbers who agreed with this statement, with 25(17.9%) respondents. This was followed by pathology and Gynecology, with 23(16.4%) and 24(17.1%) respectively. 39(27.8%) specialists disagreed with the statement that AI can be helpful for disease diagnosis accurately. Out of a total of 140 participants, 108(77.1%) expressed the belief that AI can indeed be more accurate than doctors. Among the departments, 27(19.3%) of Gynecologists showed the highest percentage of responses among other specialties who agreed with this statement. 108(77.1%) expressed the view that AI should indeed be part of the curriculum of medical students. Among the departments, radiologists showed the highest percentage of agreement with 30(21.4%) respondents.

### Nexus between acceptance of AI as future and its associated factors

In this study, multinomial logistic regression has been used to understand the impact of believing in AI’s future use on different factors. This type of regression is used in situations where there are more than two choices for an outcome. For instance, in this study, we are looking at whether specialists say “Yes,” “No,” or “to some extent” to the idea of AI being used in the future. This kind of regression helps us figure out how much these factors increase or decrease the likelihood of someone choosing each response option. In addition, the assumption of parallel lines was tested, which confirmed the appropriateness of utilizing this technique and provided a justification for its superiority over simple regression models. The model can be expressed as follows:

$$\begin{gathered}\theta \left( {Y = k|X = {x_{mi}}} \right) = logit\phi \left( x \right) =  \hfill \\\,\,\,\,\,\,\,\,\,\,\,\,ln\left[ {\frac{{\phi \left( x \right)}}{{1 - \phi \left( x \right)}}} \right] = {\alpha _{ok + }}{\alpha _{1k}}{x_{1i}} +  \ldots  + {\alpha _{nk}}{x_{ni}} \hfill \\ \end{gathered}$$

In this context, “$$ \text{Y}$$” represents the vector of the dependent variable (DV), while “$$ X$$” represents the vector of the independent variables (IVs). The number of observations is denoted by “*i*,” and “m” indicates the number of IVs. The study findings demonstrated a significant relationship between age and clinical specialists adopting AI as a future technology. Individuals under 35 years old are less inclined to accept AI (*p* = 0.019, OR = 0.049, 95% C.I; 0.004,0.609) compared to those aged 41 and above. Moreover, clinical specialists’ acceptance of AI is notably influenced by experience. Specialists with less than 6 years of experience are more likely to embrace AI (*p* = 0.0327, OR = 3.184, 95% C.I; 0.262, 3.556). Similarly, specialists with 7–11 years of experience also exhibit a higher acceptance rate (*p* = 0.261, OR = 4.095, 95% C.I; 0.351,5.754). Gender, however, does not appear to significantly impact clinical specialists' readiness to accept AI in the future (*p*-value = 0.579, OR = 1.356, 95% C.I; 0.463,3.967) (Table 4).

**Table 4:** Multinomial regression to estimate the nexus between acceptance of AI as a future and associated factors

Characteristics	Categories	*P*-value	Odds ratio	95% confidence interval for Odds Ratio	
				Lower Bound	Upper Bound
**Age**	Less than 35	0.019 ***	0.049	0.004	0.609
	36–40	0.144	0.136	0.009	1.974
	Above 41	.Reference	----	----	----
**Experience**	Less than 6	0.327	3.814	0.262	3.556
	7–11	0.261	4.095	0.351	5.754
	Above 11	.Reference	----	----	----
**Gender**	Male	0.579	1.356	0.463	3.967
	Female	Reference	----	-----	----
**AI Knowledge as a replacement in future tasks**	AI Knowledge will not replace in future tasks	0.015***	0.235	0.073	0.758
	AI knowledge will replace future tasks	.Reference	----	----	----
**AI has drawbacks and benefits**	Yes	0.084***	2.969	0.865	5.187
	No	.Reference	----	----	----
**Do you think hospitals are ready to accept AI as a new tool**	Yes	0.041***	0.172	0.032	0.928
	No	.Reference	----	----	----

*** *p*-value < 0.05 and 0.10

The last category is the reference category

Dependent Variable = Acceptance of AI as future

The study showed a significant association between the level of knowledge about AI and the preparedness of clinical specialists to embrace AI as a future technology. Specialists who firmly believe that AI knowledge will not replace their future tasks exhibit a lower likelihood of accepting AI (*p* = 0.015, OR = 0.235, 95% C.I; 0.073, 0.758). In contrast, specialists who hold a different perspective on AI’s potential impact are more inclined to accept it. Clinical specialists who perceive AI as a technology that encompasses both drawbacks and benefits demonstrate a higher likelihood of accepting its adoption (*p* = 0.084, OR = 2.969, 95% C.I; 0.865, 5.187). This characteristic exhibits a significant association with hospitals’ readiness to incorporate AI as a future technology. In essence, those specialists who recognize the nuances of AI’s advantages and challenges are more open to embracing its integration into their practices and workflows. Additionally, the overall readiness of hospitals to accept AI also shows a significant relationship with the responses of clinical specialists who express willingness to adopt AI in the future (*p* = 0.041, OR = 0.172, 95% C.I; 0.032, 0.928). This finding indicates that the hospitals’ receptiveness to AI implementation aligns with the acceptance stance of the clinical specialists within their respective organizations. When clinical specialists show a positive disposition towards AI adoption, the hospitals are more likely to be prepared and receptive to integrating AI technologies into their healthcare systems.

## Discussion

The study provides valuable insights into the perspectives of different clinical specialties regarding the potential roles of artificial intelligence (AI) in healthcare. Our study highlighted that 81(57.9%) of the participants hold the belief that hospitals are currently unprepared to incorporate AI into the future of healthcare which aligns with prior studies [9] that reported 83% of participants expressing doubts about AI’s ability to deliver empathetic patient care in the future. Moreover, our study examined participants’ grasp of AI, revealing varying levels of understanding among clinical specialists. Specifically, 54(37.1%) of the participants demonstrated above-average AI knowledge, a trend in line with recent studies [32–52]. In contrast, 41 (29.3%) of the participants exhibited an average level of AI knowledge, indicating a moderate familiarity with AI concepts. This suggests the potential value of further education and exploration of AI applications.

Concerning AI’s roles within specific specialties, the study emphasized that AI is anticipated to have a notable presence in areas such as Surgery and Pathology, particularly in the realm of documenting patient information such as health records and medical history. An exception to this pattern was observed in radiology 19(13.6%), where AI’s potential for suggesting personalized medication based on patient history stood out. Our findings are supported by prior studies [27] which indicated that 14% of radiologists agreed with the idea of AI’s role in radiology.

The integration of AI in healthcare raised significant privacy concerns among specialists, emerging as a key focus of the study. In our study, approximately 42(30%) of clinical specialists expressed apprehensions about AI’s impact on patient privacy. The outcomes are consistent with previous global studies [11, 13], although some specific studies [25, 26] diverged from this trend.

Another noteworthy concern highlighted by specialists is the fear of AI contributing to unemployment. 70 (50%) of the participants indicated worries about job loss due to AI. A similar concern was noted in a separate study [32] that shared a comparable objective but utilized a different methodology. While some studies reported differing findings, such as a study in the UAE where 64% of participants were unconcerned about AI’s negative impact on their careers [27], this fear may be influenced by the preexisting high unemployment rates in Pakistan. Addressing this fear necessitates proper planning to ensure AI complements and enhances human skills rather than fully replacing them. Considering the benefits of AI, a substantial majority, comprising 85% of participants, expressed their support for the computerization of healthcare data. This opinion was accompanied by an acknowledgment of AI’s potential in achieving precise disease diagnosis, with a significant 72.1% of respondents acknowledging this potential accuracy. A further 77.3% of participants exhibited a belief that AI’s accuracy could potentially surpass that of doctors. Additionally, a considerable 88.5% of the respondents shared an agreement on AI’s potential to alleviate the demanding workload within the healthcare sector. However, it is worth noting that our study’s findings were not uniform across all dimensions. Certain aspects, such as “Provide documentation,” “personalized medication” for patients,” and “Perform a physical examination” tasks, garnered comparatively lower percentages (17%) of agreement, as indicated by previous research [11]. A recent study conducted in Malaysia [53], which featured a participant group consisting of nearly half of physicians, demonstrated parallels with our findings. In this study, approximately 60% of the participants believed that AI would exhibit lower error rates than human physicians. In our investigation, we observed a slightly higher percentage, with 77.3% of participants holding a similar belief.

Furthermore, the study noted that 108(77.1%) of clinical specialists agreed that AI could be integrated into the medical student curriculum. Similar findings have been given in the previous studies conducted in the USA [16, 54], where a majority of doctors expressed that AI should be part of the medical curriculum.

## Conclusion

In conclusion, our study provides crucial insights into the perspectives of different clinical specialties on the potential roles of AI in healthcare. Overall, a positive attitude towards AI emerges, with many participants acknowledging its transformative impact on the future of healthcare. While reservations exist, a substantial number of specialists recognize the potential benefits and revolutionary capacity of AI in healthcare practices. There is widespread acknowledgment of AI’s diagnostic capabilities and a shared vision of its potential to enhance patient care, reduce workload, and integrate AI concepts into medical education, particularly in Surgery, Pathology, and Radiology specialties.

The study can help in providing a theoretical understanding of how healthcare professionals in developing countries adopt new technologies. It provides valuable insights that can inform existing theories on how individuals perceive and embrace technological innovations in healthcare. By exploring clinical specialists’ views on AI’s potential to replace, benefit, or disadvantage them, the study deepens our understanding of how these professionals assess and navigate emerging technologies. Additionally, its cross-sectional design enables us to analyze the relationships between various factors and AI acceptance among clinical specialists, 

As far as practical implications are concerned, the findings of the study can guide policymaking in Pakistan by providing insights into the readiness of hospitals for AI integration and highlighting concerns regarding privacy violations and potential unemployment. This information can guide the development of policies and regulations aimed at facilitating the responsible adoption of AI in healthcare systems. Secondly, understanding clinical specialists’ perspectives on AI can aid healthcare organizations in allocating resources more effectively. For instance, if a significant number of specialists express apprehensions about privacy violations, resources can be directed toward implementing robust data protection measures. Thirdly, the study underscores the need for training programs designed to equip clinical specialists with the necessary knowledge and skills to utilize AI technologies effectively. These programs can address concerns and misconceptions while emphasizing the benefits of AI adoption.

The findings not only shed light on the current perspectives of clinical specialists regarding AI in healthcare but also open avenues for future research opportunities. Especially in high-stakes fields like Radiology and Gynecology, can be a focal point for developing robust and ethical AI applications. Moreover, the study identifies a noteworthy opportunity in the apprehensions expressed by specialists regarding job stability.

## Limitations of the study

While our study has identified some significant factors related to the acceptance of AI as the future for clinical specialists, there is a significant limitation in conducting a survey study on AI in the healthcare sector specifically in the Southern Punjab region. The region faces limited access to technological resources like fast internet which could result in fewer specialists being able to respond during their job timing. Convenience sampling, while practical, poses limitations that must be acknowledged for the sake of research validity. The method may not yield a representative sample, introducing the risk of sampling bias as participants are selected based on accessibility rather than randomness. Generalizing findings beyond the specific sample and context can become challenging, compromising external validity. It is important to acknowledge that outcomes derived from this region may not generalize to other areas. The main reason is that the healthcare infrastructure in this region significantly lags behind that of other parts of Pakistan, accompanied by other factors such as facility availability, technological resources, healthcare staffing, and accessibility to advanced diagnostics and treatments.

### Electronic supplementary material

Below is the link to the electronic supplementary material.

Supplementary Material 1

## Data Availability

The datasets used and/or analyzed during the current study are available from the corresponding author upon reasonable request.
