# Supplementary material for: Exploring clinical specialists’ perspectives on the future role of AI: evaluating replacement perceptions, benefits, and drawbacks
Source: BMC Health Serv Res. 2024 May 9;24:587. doi: 10.1186/s12913-024-10928-x (PMC11080164; doi:10.1186/s12913-024-10928-x)
Supplement: Supplementary file 1 — Supplementary Material 1 [file 12913_2024_10928_MOESM1_ESM.pdf]

Serial No.

Questionnaire

Exploring Clinical Specialists' Perspectives on the Future Role of AI: Evaluating Replacement Perceptions, Benefits, and Drawbacks

Objective of the study: Over the past few decades, the utilization of Artificial Intelligence (AI) has surged in popularity, and its application in the medical field is witnessing a global increase. However, in developing nations like Pakistan, the adoption of AI-based healthcare solutions has been sluggish. This study aims to assess the opinion of clinical specialists on the future replacement of AI, its associated benefits, and its drawbacks.

1: Demographic Questions

1. What is your gender?
   (a) Male (b) Female

2. What is your age?
   ____

3. What is your total professional experience?
   ____

4. What is your professional department?
   (a) Surgery (b) Pathology (c) Radiology (d) Gynecology (e) Pediatrics

5. Do you think hospitals are ready to accept AI-driven diagnostic tools as a new tool?
   (a) Yes (b) No (c) To some extent

6. Do you think AI is the future?
   (a) Yes (b) No (c) To some extent

7. What is your level of knowledge about AI?
   (a) Poor (b) Average (c) Above Average (d) Excellent

2: Tasks that AI can Perform in the Future as a replacement for Specialists

This sub-scale consisted of nine questions consisting of AI's different roles as replacements of documentation, clinical care to patients, suggesting medication, conducting a physical examination, diagnosis, and patient history. Please tick(✓) only one option for each question.

Questions	Possible	Not Possible	May be Possible
Is it possible that AI can be helpful in Documentation about patients (Health records, patient history, etc)?			
Is it possible that AI can be helpful in providing good care to patients?			
Is it possible that AI can be helpful in suggesting personal medication based on the patient's history?			
Is it possible that AI can be helpful in evaluation of the patients for treatments?			
Is it possible that AI can be helpful in examining patient information to establish prognoses?			
Is it possible that AI can be helpful in employing advanced techniques to detect instances of self-harmful behavior in patient information			
Is it possible that AI can be helpful in conducting a comprehensive physical examination, encompassing a detailed mental status assessment			
Is it possible that AI can be helpful in utilizing advanced methods to discern potential indications of criminal behavior or assault in patient information			
Is it possible that AI can be helpful in conducting patient interviews across diverse settings to gather their comprehensive medical history			

3: Opinion of Specialists about the Benefits and Drawbacks of AI

This sub-scale consisted of eight questions about AI benefits and drawbacks including the concerns about violation of patients' privacy, unemployment issues, reducing the work and paper burden, help for specialists, and part of the curriculum. Please tick(✓) only one option for each question.

Questions	Yes	No	May be
Do you think that AI can be a violation of patients' privacy?			
Do you think that AI can cause unemployment?			
Do you think that the computerization of Healthcare data of patients can offer an opportunity to improve patient care?			
Do you think that the utilization of AI can substantially minimize the paperwork burden of keeping records of Patients?			
Do you think that AI can reduce the burden of work?			
Do you think that AI can be helpful for disease diagnosis accurately?			
Do you think that AI can be more accurate than doctors?			
Do you think that AI can be part of the curriculum of Medical students?			

Ethics approval

This study adhered to the Declaration of Helsinki for the recruitment of human subjects and was approved by the District Health Department, Human Ethical Committee of Multan, Pakistan. Informed consent was obtained from all the participants and/or their legal guardians.

Privacy Statement

Your privacy is important to us. This survey has been designed to collect information for research purposes only. All responses are completely anonymous, and no personally identifiable information will be linked to your answers. The data collected will be used solely for research, and findings may be shared in aggregated, non-identifiable form. Any personal information provided will be kept confidential and separate from your survey responses. Participation in this survey is entirely voluntary, and you can withdraw at any time without penalty.
